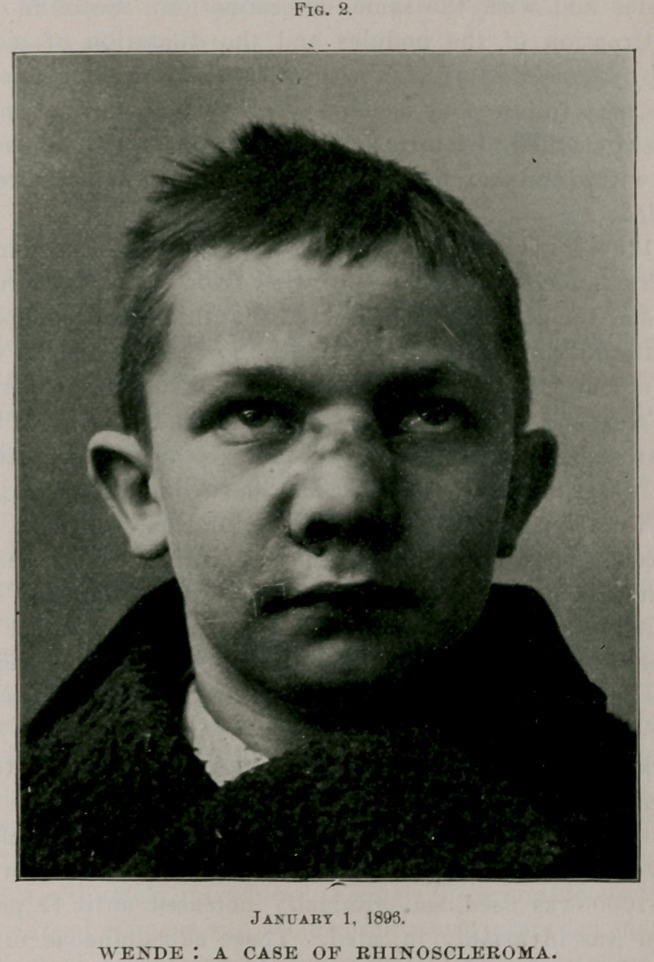# A Case of Rhinoscleroma Originating in the United States1Read at the meeting of the Pathological Section of the Buffalo Academy of Medicine, January 21, 1896.

**Published:** 1896-03

**Authors:** Grover William Wende

**Affiliations:** Clinical instructor of skin diseases, University of Buffalo, physician for diseases of the skin at the Erie County Hospital


### A CASE OF RHINOSCLEROMA ORIGINATING IN THE UNITED STATES.[1]

By GROVER WILLIAM WENDE, M. D.
Clinical instructor of skin diseases, University of Buffalo, physician for diseases of the skin at the Erie County Hospital.

IN THE year 1870, Hebra and Kaposi, for the first time, conjointly described the exceedingly rare affection of the skin which they designated as rhinoscleroma.

It was characterised by Kaposi as a new growth, closely allied to sarcoma, with its seat about the nose, occurring usually on the septum or upon one or both alæ. There was no pain, nor were there any symptoms of an inflammatory nature present. The initial lesions were nodules embedded in the cutis and in the deeper layers of the mucous membrane. The parts thus involved slowly enlarged, increased in density, and eventually felt like ivory to the touch, extending upward from the lip and downward to the pharynx from the posterior nares. Individually, these manifestations were flat plaques or nodules elevated and circumscribed in appearance. They were at times papular and tubercular. When pressed they were painful. This new growth involved the skin and could only be moved with it. However, there was no attachment to the underlying structure—bone and cartilage remaining free. No hair or glands were discernible in these lesions.

As the disease progressed, the alæ became enlarged, flattened and indurated to such an extent that they could not be pressed together. Apart from the deformity, the pain on pressure, the interference with respiration, and finally the danger of death from suffocation, the general health was unimpaired.

Geographically, I found upon reference that rhinoscleroma was not uniformly distributed: Austria leading with 63 cases, being nearly one-half of all cases recorded; next came South West Russia,

---

1. Read at the meeting of the Pathological Section of the Buffalo Academy of Medicine, January 21, 1896.

to which are credited 37; then followed Central America, 24; Italy, 7; Burmah, 4; Egypt, 2; Brazil, 3; England, 3; France, 2, and Buenos Ayres, 1.

In this country, according to reports of the American Dermatological Association, only seven cases were placed upon record,

FIG. 1.

SEPTEMBER 1, 1895.

WENDE: A CASE OF RHINOSCLEROMA.

and in every instance these were of foreign origin, emanating usually from Austria, where the tumors first appeared.

CASE.—The patient, whom I have the honor to present before this Academy for its consideration, was referred to me, four months ago, by Dr. Roswell Park. He is of American parentage, a resident of Buffalo, the place of his birth, which he never left but on one occasion, and that was on December 18, 1895, when he was presented before the New York Dermatological Society. He is 11 years of age, and is apparently strong and robust. His family history is exceptionally good, exclusive of the mother who died at the age of 33 during pregnancy. His grandparents were noted for their remarkable longevity, his paternal grandfather having attained the age of 90, and his paternal grandmother having exceeded the age of 101 years; while on the maternal side their respective ages were 80 and 78. The father, a locomotive engineer, is 44 years old and is seemingly a typical specimen of health and strength. He has two brothers, aged respectively 17 and 18, who have always enjoyed the best of health.

It was ascertained upon inquiry that the condition from which the patient was suffering had existed for about a year and a half, though in a less marked degree, and was progressive in its nature. He disclaimed all knowledge of any injury or exposure to irritating influences of any description. His father was the first to observe the change in the normal skin, consisting then of a pink spot slightly raised, below the right nares. There was no pain. At the expiration of three months a perceptible elevation appeared, the beginning of what soon developed into a pronounced ridge.

These manifestations, the spot and the ridge, were joined at their margins near their respective centers. As time went on, the ridge assumed greater proportions and finally extended to the left side of the nose.

My first examination revealed a nodule, irregular in outline, just below the right nares, as seen portrayed in the accompanying illustration, Fig. 1—which was removed sometime in August for a microscopical investigation. Its structure was quite superficial, while the tissue beneath was infiltrated and very hard. Upon the right side of the nose two sharply-defined ridges were seen, each measuring about one and three-fourths inches in length, having between them areas of unaffected skin. The upper one was the least prominent, and was of uneven width.

The left side of the nose showed but one ridge, which had a uniform width of nearly half an inch. These ridges, which were exceedingly pronounced and indurated, united upon the bridge of the nose. The right ala was uniformly thickened, causing a narrowing of the corresponding lumen of the nostril. The left ala, at this time, was not involved; the line of demarcation between these lesions and the healthy skin was abrupt.

In considering the diagnosis we may readily eliminate rhinophyma, tubercular lepra, tubercular lupus, keloid, epithelioma and sarcoma by the process of exclusion. That it is not syphilis has been demonstrated by the fact that a thorough antisyphilitic treatment for one year proved fruitless.

From the disfigurement, its glossy appearance and localisation, its origin from the nasal mucous membrane, later, its encroachment upon the lower part of the nares, its extension backward in the nasal cavity to the posterior nares, its gradual development without disintegration, its peculiar hardness and elasticity, its extension to the upper lip with a sharp border and its regeneration of the excised

FIG. 2.

JANUARY 1, 1896.

WENDE: A CASE OF RHINOSCLEROMA.

portions,—all this has led me to conclude that my patient is afflicted with no other disease than rhinoscleroma. The case is one of unusual interest, not alone for its rarity, but from the fact that it is the first appearance of the affection in an American by birth ever recorded.

The treatment of this disease is most unsatisfactory, as the growth is liable to return almost immediately after the removal.

All that can be accomplished is merely palliative—namely, keeping the air-passages open as far as practicable by dilatation through the introduction of catgut drainage-tubes or compressed sponges.

To establish a cure, cauterisation with the galvano-cautery, caustic potash, pyrogallic acid, or the total extirpation with subsequent plastic operation, have been recommended on the same principles and with the same determination. Secretan advised the extirpation of the nodules and the dilatation of cicatricial bands by means of intubation with Schrötters' sound. This course, he says, was followed by decided improvement, the patient being afterward enabled to return to work. However, the plaques reappeared within one year, but the tissue, relaxed by dilatation, remained distended.

In 1894, Pawlowsky, of Kiew, treated two cases hypodermically with rhinosclerin, an extract prepared from a pure culture of the bacillus of the disease. He observed that the formation of cultures was materially modified by the addition of the extract to the culture medium. He further demonstrated that an injection of the glycerin extract of the bacillus into a patient 18 years old induced fever, swelling, and redness of the nose. One month later, after fifteen injections, the process became so subdued that the plaques, which were so prominent in their induration, softened, and on examination showed symptoms of a condition which was of an inflammatory nature. After the expiration of one year's treatment, its intensity was arrested and all progress ceased. Thus Pawlowsky ventures to hope that he has discovered a remedy of diagnostic and therapeutic value in the treatment of rhinoscleroma by injecting rhinosclerin.

Stuknovenkoff, of the same city, puts his faith on the subcutaneous medication with arsenic, which was employed by him in a patient aged 21 years, who had suffered from this malady for three years. For the first four days a mixture of Fowler's solution 1-1000 was used, and gradually increased until 12 per cent. solution was invariably injected. These subcutaneous injections were continued for fifteen months, their number amounting to 200 in all. There was at once an apparent alleviation of symptoms and a disappearance of the new growth. Six months subsequent to the last injection the patient gave no evidence of relapse; improvement seemed permanent and his general health good.

Encouraged by the result obtained by Stuknovenkoff, I decided it no longer desirable to consider the treatment of rhinoscleroma as essentially palliative; therefore I have now begun injecting a solution of arsenic into the plaques of the disease of my patient. I shall reserve judgment for a while as to its probable result. At the same time it is fair to say that I am not altogether hopeful as to its real and permanent value.

An account of the examination of the excised tissues by Dr. Herbert Williams will appear later in the JOURNAL.

471 DELAWARE AVENUE.

## Figures and Tables

**Fig. 1. f1:**
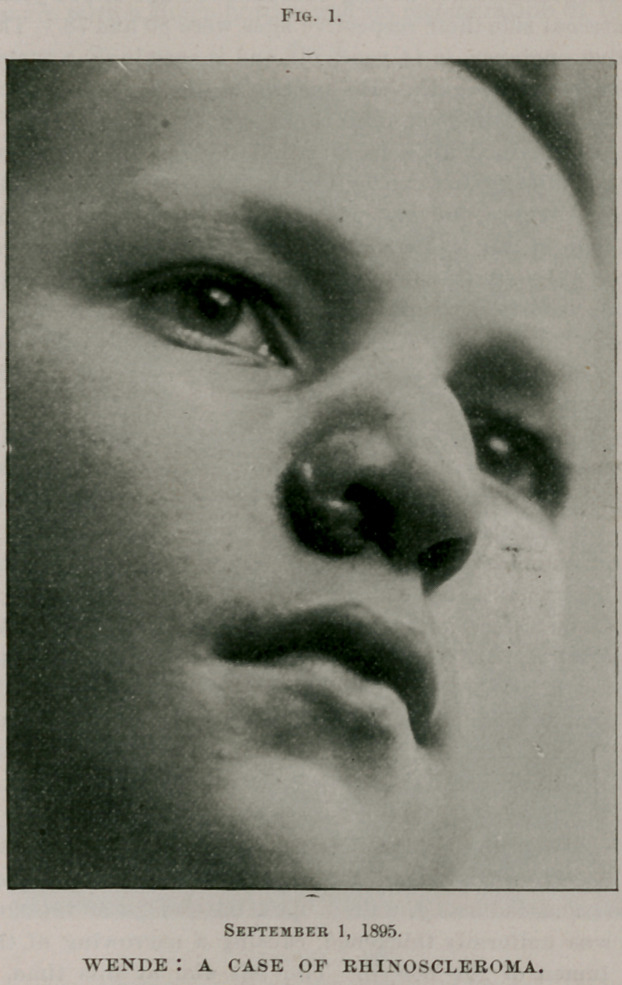


**Fig. 2. f2:**